# Context is key: Maternal immune responses to pig allogeneic embryos

**DOI:** 10.1002/mrd.23624

**Published:** 2022-06-26

**Authors:** Cristina A. Martinez, Heriberto Rodriguez‐Martinez

**Affiliations:** ^1^ Department of Biomedical & Clinical Sciences (BKV), BKH/Obstetrics & Gynaecology, Faculty of Medicine and Health Sciences Linköping University Linköping Sweden

**Keywords:** allogeneic embryos, establishment of pregnancy, maternal immune rejection, pig, pregnancy

## Abstract

Successful establishment of pregnancy includes the achievement of a state of immune tolerance toward the embryos (and placenta), where the well‐coordinated maternal immune system is capable of recognizing conceptus antigens while maintaining maternal defense against pathogens. In physiological pregnancies, following natural mating or artificial insemination (AI), the maternal immune system is exposed to the presence of hemi‐allogeneic embryos, that is, embryos containing maternal self‐antigens and foreign antigens from the paternal side. In this scenario, the hemi‐allogeneic embryo is recognized by the mother, but the immune system is locally modified to facilitate embryo implantation and pregnancy progression. Pig allogeneic pregnancies (with embryos containing both paternal and maternal material foreign to the recipient female), occur during embryo transfer (ET), with conspicuously high rates of embryonic death. Mortality mainly occurs during the peri‐attachment phase, suggesting that immune responses to allogeneic embryos are more complex and less efficient, hindering the conceptuses to survive to term. Reaching a similar maternal tolerance as in conventional breeding would render ET successful. The present review critically summarizes mechanisms of maternal immune recognition of pregnancy and factors associated with impaired maternal immune response to the presence of allogeneic embryos in the porcine species.

## 1 INTRODUCTION

Embryo–maternal dialog is crucial to achieve maternal recognition and endometrial receptivity during the peri‐implantation period of early pregnancy (Waclawik et al., 2017). During this period, porcine conceptuses modulate mechanisms of the maternal environment through the secretion of different molecules such as hormones, growth factors, and cytokines, establishing a balance between pro‐and anti‐inflammatory signals facilitating successful pregnancy (Bazer et al., 2010). Under physiological conditions, the embryo is considered hemi‐allogeneic to the mother since it contains paternal antigens that are foreign to the maternal immune system. Maternal recognition of the hemi‐allogeneic embryo occurs by downregulating immune signals to avoid embryo rejection and favor the establishment of pregnancy, while maintaining immune capacity against pathogens (Mor et al., 2010; PrabhuDas et al., 2015). It has been suggested that allogeneic embryos, that is, embryos containing both paternal and maternal material differing from that of the recipient, induce a less efficient maternal immune response, triggering embryo mortality. As it has been widely stated, porcine embryo transfer (ET) is burdened by high rates of embryonic loss and deficiencies in embryonic development (Martinez et al., 2019, 2020), mainly during the peri‐implantation period of pregnancy. Research carried out across a wide range of species has focused on the interaction between the conceptus and the maternal reproductive tract, as well as how conceptus‐derived factors interact with the uterus to establish an adequate environment for pregnancy. However, little is known about the mechanisms involved in the communication between the maternal interface and the allogeneic embryo during the peri‐implantation period; mechanisms that would have relevant scientific and economic impacts both for livestock and human.

The purpose of this review is to summarize recent progress toward the understanding of maternal recognition and the establishment of pregnancy following artificial insemination (AI)‐ or ET‐pregnancies in pigs, with a focus on what could provoke failure and thus pointing out possible strategies to diminishing embryo death after ET.

## 2 ESTABLISHMENT OF PREGNANCY: EMBRYO–MATERNAL CROSSTALK

To establish pregnancy and support embryo and placental development, porcine conceptuses release multiple factors during the peri‐implantation development and regulation of the maternal immune system. Several studies have examined the role of estrogens (E_2_), prostaglandins (PG), interleukin 1 beta 2 (IL1B2), and interferons (IFNs) derived from the conceptus, suggesting they play key roles for the maternal recognition of pregnancy, the stimulation of endometrial secretions to support embryo growth and development, and of the maternal immune regulation (Geisert, 2015).

Estrogen production by the conceptus has been widely proposed and accepted as the main signal for porcine maternal recognition (Meyer et al., 2019). Inhibition of luteolysis and the attachment of trophectoderm to the uterine epithelial lining were determined as dependent on biphasic increases in E_2_ synthesis on Days 11 and 15–30 of pregnancy (Geisert et al., 2006). In addition, E_2_ helps to promote the migration of embryos and their equidistant spacing along the uterine horns before rapid elongation and attachment of the conceptus to the endometrium (Geisert et al., 2017). E_2_ further stimulates the transcription of a large number of endometrial genes responsible for growth factors, attachment and adhesion proteins, prostaglandin synthesis, receptor signaling, ion transport, and transporters of glucose and amino acids (Ka et al., 2018). In addition to its role in CL maintenance (Waclawik, Blitek, et al., 2009; Waclawik, Jabbour, et al., 2009), the synthesis of prostaglandin by the pig conceptus has also been implicated in endometrial function (Waclawik, Blitek, et al., 2009; Waclawik, Jabbour, et al., 2009). When prostaglandin synthesis is inhibited during early pregnancy, conceptus loss occurs (Mayoral Andrade et al., 2020), suggesting their central role in early embryonic development and survival.

Production of IL1B2 by the pig conceptus is also an essential factor for cellular remodeling of the trophoblast during the period of rapid elongation and survival. The expression of IL1B2 occurs during rapid trophoblast elongation (Geisert et al., 2017; Zhao et al., 2014) acting through its specific receptor (IL1R1) to initiate molecular signaling pathways through activation of nuclear factor kappa‐B (NFKB) in the epithelial cells lining the endometrium (Mathew et al., 2015), a component of the immune system that senses and adapts to alterations in the tissue's microenvironment.

Immediately following trophoblast elongation (Day 12 of pregnancy), the pig conceptuses express both Type I (IFND) and Type II (IFNG) interferons which remain detectable until Day 20 of pregnancy (Bazer & Johnson, 2014; Bazer et al., 2009).

Pig conceptus trophectoderm is unique in secreting both IFND and IFNG, which act through receptors IFNAR1 and IFNGR1, respectively; playing important roles in conceptus implantation through their involvement in regulating integrins and heparin sulfate proteoglycans, as well as contributing to the remodeling of the endometrial epithelium, affecting its polarity and receptivity to the trophoblast; processes that promote embryo attachment (Imakawa et al., 2018).

## 3 IMMUNE RESPONSES AT THE EMBRYO–MATERNAL INTERFACE

A delicate immunological balance at the embryo–maternal interface is necessary for proper embryo development, maintenance of pregnancy, and to prevent immune embryo rejection (Sinkora et al., 2009). In this regard, conceptus‐derived factors are responsible for maintaining an adequate immune balance. IFNG is shown to regulate a large number of endometrial genes involved in immune tolerance during the process of conceptus attachment to the luminal epithelium and placental development (Johns et al., 2021; Yoo et al., 2020). Conceptus IFNG is considered responsible for inducing the expression of swine leukocyte antigen‐ DQA (SLA‐DQA), a major histocompatibility complex (MHC) class II gene, in pig endometrium at the time of conceptus implantation; likely regulating immune response at the maternal‐fetal interface to support the maintenance of pregnancy (Kim et al., 2012). Moreover, IFNs produced by the conceptus induce the expression of endometrial interferon‐stimulated genes (ISGs) which in turn, trigger the establishment of a uterine vascular supply to the conceptus, cellular homeostasis, and physiological adaptations, facilitating embryo tolerance and implantation (Austin et al., 2003; Bany & Cross, 2006; Hess et al., 2007). Cytokines, which modulate innate and adaptive immune systems by acting locally in an autocrine/paracrine manner, are also induced by conceptus‐derived factors and play a crucial role in cellular and molecular events related to endometrial adaptation for embryo encounter as well as elongation and embryo development, thus promoting an adequate establishment of pregnancy. They also participate in many physiological events such as inflammation, angiogenesis, and endometrial remodeling (McLendon et al., 2020). These endometrial vascular changes coincide with the recruitment of immune cells, like natural killer cells, T cells, dendritic cells, and macrophages to the site of embryo attachment (Chu et al., 2021; Croy, Wessels, Linton, & Tayade, 2009). These recruited immune cells actively contribute to the regulation of placental development, homeostasis, and tolerance of the fetal allograft. Dendritic cells process and present antigen to T cells via MHC to subsequently participate in those tissue remodeling and vascular changes apparently necessary to support the attachment of trophoblast cells to the endometrium (Linton et al., 2008).

Through these mechanisms, both innate and adaptive maternal immune systems are competent to sense both hemi‐allogeneic conceptus‐derived and external environmental factors and accordingly influence the progression of pregnancy.

## 4 SPONTANEOUS EMBRYO LOSS IN PHYSIOLOGICAL PREGNANCIES

Spontaneous embryo loss remains a significant problem for the porcine industry worldwide. The highest rate of embryo death occurs during the peri‐implantation period of pregnancy (Days 12–30 of pregnancy; Bidarimath et al., 2017; Edwards et al., 2012). Recent research has attempted to identify and clarify the causal relationship between spontaneous embryo loss and various risk factors (Bazer & Johnson, 2014; Hunter et al., 2020; Kridli et al., 2016). Many factors could motivate the rejection of existing conceptus tissue—for example when injury or infection of the reproductive tissues occur, or in the presence of chromosomal deviations, lack of uterine capacity, nutrition, environment, placental condition, or immune mechanisms among others (Bertoldo et al., 2009; Croy, Wessels, Linton, Heuvel, et al., 2009). Maintenance of pregnancy requires an equilibrated balance of inflammatory responses to promote an increase in vascularity and endometrial remodeling without causing conceptus loss. Interestingly, when the embryos are subjected to any detrimental condition like the ones mentioned above, they release danger signals recognized by the maternal immune system, activating a cascade of immunological events toward embryo death (Bidarimath et al., 2017). Thus, compromised or altered trophoblast secretory function is likely to alter maternal immune response, potentially causing immune rejection probably by modifying dendritic cell influence on angiogenesis and placental access to the maternal blood supply (Blois et al., 2007; Laskarin et al., 2007).

Similar effects are induced by the altered cytokine balance associated with detrimental conditions. In this regard, inadequate expression of pro‐inflammatory cytokines (such as tumor necrosis factor‐alpha [TNF‐α] and IFNG) at the implantation site has been associated with increased embryo loss in pigs compared to normal pregnancies (Bidarimath et al., 2016). Whether the localized increase in pro‐inflammatory cytokines at the site of embryo attachment is the cause or the consequence of embryo loss is not yet totally clear.

Previous studies of the changes in pro‐inflammatory cytokine profiles during the peri‐implantation period of pregnancy revealed an interesting association between changes in the expression of cytokines such as TNF‐α, IFNG, tumor necrosis factor alpha‐inducible protein 6 (TNFAIP6), and IL6, among others, and augmented embryo loss (Ali et al., 2021; Robertson et al., 2018). Specifically, as a result of abnormal estrogen levels, a decreased Inter‐Alpha‐Trypsin Inhibitor Heavy Chain 2 (ITIH2) can be implicated in the disruption of glycocalyx and/or the remodeling of the extracellular matrix, either potentially leading to conceptus degeneration (Ashworth et al., 2010). As well, significantly increased IFNG endometrial levels on Days 16–18 of pregnancy have been linked to increase embryo loss (Martinez et al., 2020), whereas fetal loss during mid‐gestation (Day 50) was not influenced by the expression of this cytokine (Tayade, Fang, & Croy, 2007), suggesting that different mechanisms and temporal changes over the course of pregnancy may contribute to embryo/fetal loss. Moreover, TNF‐α, IL1B, and IL1 receptors were all found upregulated in the endometrial attachment sites of arrested embryos, suggesting that an acute inflammatory reaction is detrimental for embryo survival (Tayade, Fang, Hilchie, et al., 2007). The expression of several other cytokines has been explored in the site of implantation of arrested or degenerated embryos and compared to their levels in healthy embryo attachment sites. A higher abundance of CXCR3, CCR5, CXCL10, and CCL5 was identified at arresting conceptus attachment sites by Day 20 of pregnancy, as affecting immune cell recruitment and placental function (Bidarimath et al., 2016).

On the other hand, restricted growth and subsequent embryonic death are associated with impaired vascular development at the maternal‐fetal interface (Croy, Wessels, Linton, & Tayade, 2009). Endometrial vascular remodeling around the time of implantation is essential to provide nutrient supply to the conceptus (Lee et al., 2017). A signal protein known as vascular endothelial growth factor A (VEGF‐A), promotes blood vessel formation, thus remodeling the endometrium's vasculature (Kaczmarek et al., 2008). Porcine endometrial stromal cells transcribe VEGFA in response to Insulin‐like growth factor 1 (IGF1) and Prostaglandin E2 (PGE_2_) during the pre‐implantation period, while conceptus‐derived VEGFA levels gradually increase until implantation occurs (Waclawik, Blitek, et al., 2009; Waclawik, Jabbour, et al., 2009).

## 5 PREGNANCY OUTCOMES AFTER ALLOGENEIC ET

The technology to achieve successful pregnancies after the transfer of embryos (ET) into the sow's reproductive tract has improved over the past decade (Martinez et al., 2019). ET has not only been helpful to increase our knowledge of fundamental processes of reproduction but has also become relevant for pig breeding through the transfer of genetically valuable embryos with extremely low risk of disease transmission, reduced transportation costs, and preservation of animal welfare, or even serving many biotechnologies such as somatic cell cloning or gene editing. However, the use of ET by the pig industry is still limited by the high embryo mortality associated with either surgical or nonsurgical ET, precluding their application in the field.

Surgical ET relies on the transfer of embryos into the oviduct or the tip of a uterine horn, depending on the stage of embryo development. This procedure has been optimized and re‐evaluated over the past years (Martinez et al., 2017). The best pregnancy outcomes (pregnancy rates of about 60%–80% with an average litter size of 7–8 piglets) using surgical ET have been reached after the transfer of Days 3–8 embryos into recipients that exhibited estrus 1–2 days after donors (Martinez et al., 2016). However, this technique has a very limited practical application due to the need, among others, of surgical facilities, anesthesia, and equipment; prerequisites to maintain animal welfare.

**Figure 1:**
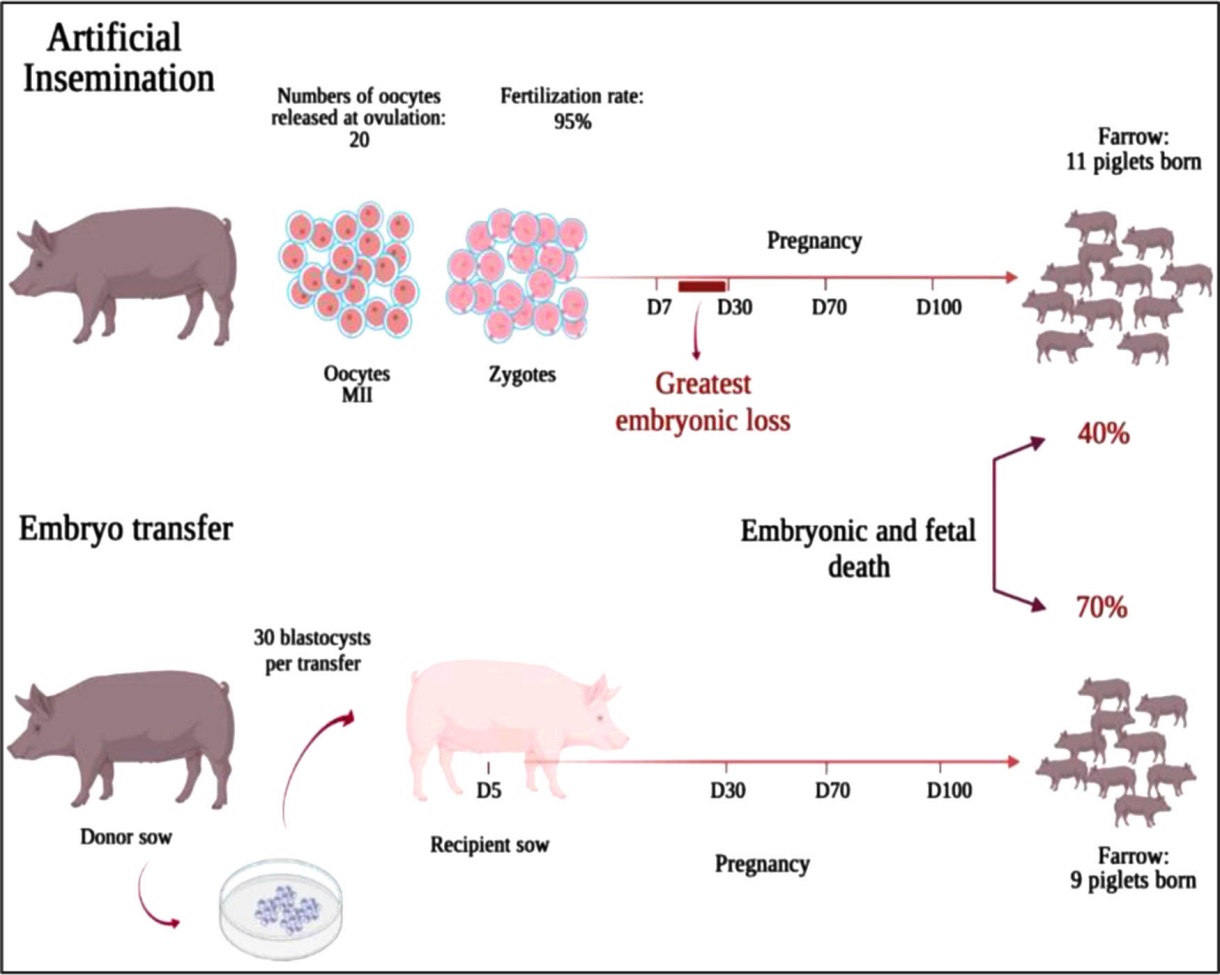
Schematic representation of pregnancy outcomes after artificial insemination or nonsurgical embryo transfer

Early development of nonsurgical procedures for ET proved to be successful in producing viable piglets after transfer of in vivo‐derived embryos. Since then, many researchers have developed and improved new protocols using specially designed catheters (Nohalez et al., 2015). Up to date, the nonsurgical deep uterine (NsDU)‐ET technique, involving the insertion of a flexible and thin device into a uterine horn through a catheter that is able to progress along the uterine lumen to the site of embryo deposition, appears to be the technique with best pregnancy outcomes when using 30–40 fresh or stored embryos (Angel et al., 2014; Martinez et al., 2004, 2014, 2015). However, its efficiency is still lower than the surgical approach or AI (Figure 1). Approximately 70% of the transferred embryos are lost during pregnancy, mainly during the period of peri‐implantation (Martinez et al., 2020).

Many factors might contribute to pregnancy failure both in surgical and nonsurgical ETs: recipient condition, type of protocol for ET, embryo origin, embryo deposition site, volume of transfer medium associated with the embryos, among others. Yet, little attention has been paid to the fact that ET embryos are foreign to the recipient both on the maternal and paternal sides, for example, containing maternal and paternal antigens not to be recognized by the maternal immune system when deposited into the recipient's reproductive tract, ultimately favoring immune embryo rejection and embryo death.

## 6 POSSIBLE MECHANISMS TRIGGERING MATERNAL IMMUNE REJECTION IN PIG ALLOGENEIC PREGNANCIES

The capacity of the maternal immune system to recognize embryo‐derived signals and exert tolerance or rejection of embryos suggests a key role of the female immune response in its responsibility to establish or reject pregnancy, the “intrinsic female choice” (Ander et al., 2019). Extensive research has pointed out the coordinated role of innate and adaptive maternal immune responses to distinguish self from non‐self in hemi‐allogeneic pregnancies, for example, after natural mating or AI, where the maternal immune system is programmed to “ignore” paternal alloantigens since the immune system has been exposed to paternal antigens in seminal fluid from the moment of conception (Moldenhauer et al., 2009), thus providing a continuous influx of antigenic material that persists systemically in the mother along pregnancy (Erlebacher et al., 2007).

**Figure 2:**
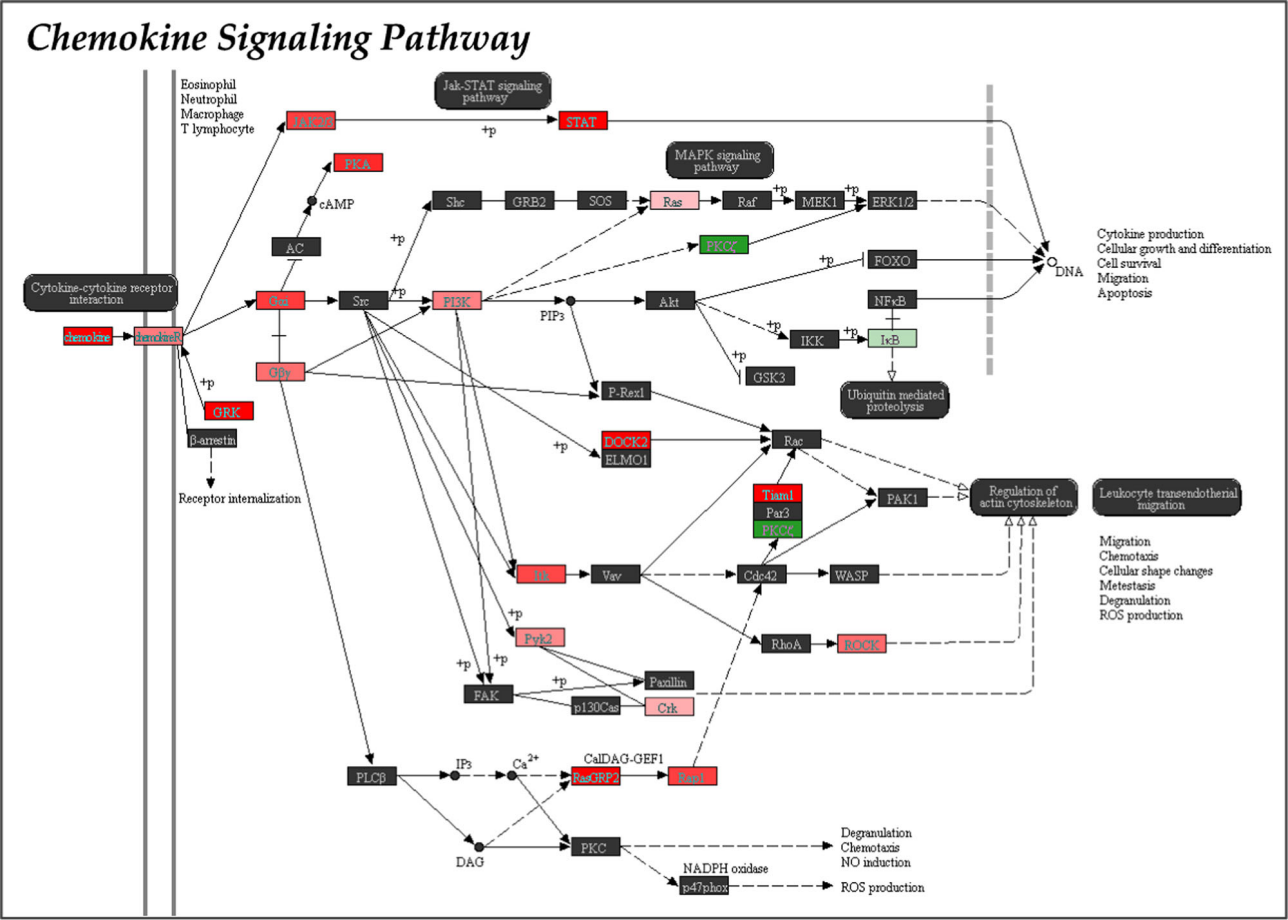
Schematic representation of “Chemokine Signaling” pathway. Downregulated genes (*p* < 0.05) in endometrial samples from embryo transfer sows compared to sows subjected to artificial insemination are red‐marked. Upregulated genes are green‐marked. Color intensity represents the degree of down‐ or upregulation.

A more complex interplay takes place in the case of allogeneic‐ET pregnancies, where both maternal and paternal antigens are foreign to the recipient. Unfortunately, information on the response of the maternal immune system to the stimuli produced by allogeneic embryos in livestock is scarce. Our group recently reported that the transfer of allogeneic pig embryos leads to a significant increase of embryo loss and a conspicuous delay in embryo development during the peri‐implantation period, compared to hemi‐allogeneic embryos (Martinez et al., 2020). The possible causes for these results and its association with maternal immune responses were investigated throughout several studies performed by our group in the last few years. We first studied the changes in the expression of pro‐ and anti‐inflammatory cytokines in response to the presence of allogeneic embryos (after ET) compared to hemi‐allogeneic pregnancies during the peri‐implantation period of pregnancy (Days 18 and 24 of pregnancy). Several pro‐inflammatory cytokines with roles in uterine receptivity, maternal immune tolerance, and vascular changes essential to nourish the developing embryos (endometrial IL‐2 and IFN‐γ, and placental IL‐2 and TNF‐α), were repressed in the allogeneic endometrium, presumably suggesting that lack of conceptus signaling might not stimulate an appropriate inflammatory response in the endometrium (Martinez et al., 2020). Additionally, transforming growth factor‐beta 2 (TGF‐β2), an anti‐inflammatory cytokine with pivotal roles in angiogenesis, immunotolerance, embryogenesis, embryo attachment, and tissue remodeling, was downregulated in allogeneic endometria at Day 18 over Day 24, suggesting its implication in the disruption of cell adhesion or as a sign of immune maternal rejection during the peri‐implantation period. Furthermore, interleukin IL‐10, which significantly participates in gradually switching the pro‐inflammatory environment to an anti‐inflammatory state at the maternal‐fetal interface and helps stimulation of angiogenesis, was significantly lower in allogeneic endometrium when compared to hemi‐allogeneic endometria at Day 24, probably leading to a failure in suppressing the immune response, thus favoring allograft rejection (Martinez et al., 2020). The same pattern was observed when we analyzed gene and protein expression of leukemia inhibitory factor (LIF), known to induce differentiation of endometrial macrophages into an anti‐inflammatory/regulatory phenotype by inhibiting the signaling pathways STAT‐1 and STAT‐5 (mediated by IFNγ and GM‐CSF activation) and by promoting the anti‐inflammatory pathway STAT‐3, and in many other genes involved in the chemokine signaling pathway (Figure 2). Lower levels of cytokines/chemokines, as we observed in the allogeneic endometrium, might result in failure to create an anti‐inflammatory environment necessary to protect the embryos against immune rejection (Cambra et al., 2020).

In a more recent study, we found a conspicuous downregulation of many transcripts potentially involved in the regulation of immune responses in endometrial tissue in the presence of allogeneic embryos in comparison with hemi‐allogeneic embryos during the peri‐implantation period of pregnancy (Martinez et al., 2022). The low number of embryos found at this point in the reproductive tract, in the case of allogeneic pregnancies, or an inefficient signal on their behalf, could trigger inadequate maternal immune responses compromising further embryo development and implantation. In this report, several genes described as ISGs, stimulated by conceptus estrogen and/or IFNG and IFND (Fleming et al., 2009; Joyce, Burghardt, Geisert, et al., 2007; Joyce, Burghardt, Hooper, et al., 2007) were downregulated in the allogeneic group at Day 24 of pregnancy in comparison to the hemi‐allogeneic group (CXCL10, CXCL8, IRF1, IRF9, STAT1, etc.). Chemokine ligand 10 (CXCL10) is an ISG ruling relevant endometrial functions; including recruitment of immune cells to the implantation site or promoting attachment of the trophectoderm to the epithelium lining of the endometrium (Dufour et al., 2002). Previous reports have pointed out that an increase in the expression of CXCL10 mRNA in the pig endometrium is a pregnancy‐dependent event that takes place during early pregnancy (Gray et al., 2006; Imakawa et al., 2006; Sakumoto et al., 2017, 2018), with potential to attract embryos to the attachment site (Dominguez et al., 2008; Imakawa et al., 2005). Recently, Złotkowska et al. (2019) reported a significant increase in the mRNA expression of CXCL10 in the porcine endometrium during the peri‐implantation period, suggesting their involvement in the processes of establishing an adequate environment for the embryo by regulating immune cell recruitment and redistribution. Repression of the ISG CXCL8 (Chemokine ligand 8) at Day 24—endometrial tissue was also observed in allogeneic compared to hemi‐allogeneic pregnancies. An adequate expression of CXCL8 is essential for the formation and proliferation of capillary‐like structures (Singh et al., 2011) and angiogenesis, prerequisites for further embryo nutrition and placenta function (Złotkowska et al., 2019). The repression of CXCL8 found in that study in the allogeneic group could contribute to explaining the delay in embryo development due to the lack of vascularity observed in those conceptuses retrieved at Day 24 of pregnancy, as described in a previous report from our group (Martinez et al., 2020). Other important ISGs were also repressed in allogeneic endometrium: IRF1, IRF9, STAT1, and B2M. Their expression is known to increase in the pregnant endometrium during the peri‐implantation period in healthy pregnancies in several species, helping to stimulate embryo growth, endometrial vascularity support, and angiogenesis (Johnson et al., 2009; Joyce, Burghardt, Geisert, et al., 2007; Joyce, Burghardt, Hooper, et al., 2007; Liu et al., 2021; Musavi et al., 2018; Shirozu et al., 2016).

Likewise, the signal transduction and activator of transcription 1 (STAT1) was found downregulated in the allogeneic endometrium at Day 24 of pregnancy. STAT1 is regularly upregulated in the stroma of the ruminant uterus during pregnancy in response to IFN signaling (Carvalho et al., 2016; Liu et al., 2021; Stewart et al., 2002) and also in the pig endometrium from Day 15 of pregnancy (Johnson et al., 2009), impacting positively both uterine receptivity and conceptus implantation and development (Joyce, Burghardt, Geisert, et al., 2007; Joyce, Burghardt, Hooper, et al., 2007).

In addition, at Day 24 of pregnancy, several genes involved in the process of antigen presentation (B2M, ERAP1, ERAP2, and RAB8B) were downregulated in the allogeneic group compared to the hemi‐allogeneic group. Endoplasmic reticulum aminopeptidase 1 and 2 (ERAP1/ERAP2) are zinc‐metallopeptidases involved in the regulation of antigen presentation by MHC molecules located on the cell surface (Rastall et al., 2014). An adequate process of antigen presentation is essential for promoting an adequate immune response during pregnancy to induce trophoblast invasion, tissue remodeling, embryonic development, and placentation (Zhang et al., 2021). In response to all these altered signals, maternal immune cells can reduce or even stop the transcription of angiogenic factors, blocking vascularization of the yolk sac and particularly the allantois; leading to inhibition of conceptus development and death (Samardžija et al., 2020).

## 7 CONCLUDING REMARKS

In conclusion, porcine ET‐pregnancies imply a complex modulation of the maternal immune system which is inefficient in avoiding the rejection of allogeneic embryos, probably due to an inappropriate immune recognition of the allograft antigens; thus leading to embryo arrest and death of a high proportion of the transferred embryos. Although the key players at the maternal‐fetal interface require further exploration, the investigations summarized in the present review highlight the complexity of molecular interactions between the endometrium and the embryo influencing embryo survival, implantation, and development. This will hopefully contribute to a better understanding of the biological significance of immune system processes after the transfer of allogeneic embryos in the context of embryo and fetal loss, prerequisites for devising new strategies to improve embryo survival in porcine ET programs, a matter of great interest to livestock species and human.

## CONFLICT OF INTEREST

The authors declare no conflicts of interest.
